# Divalent Metal Ion Depletion from Wastewater by RVC Cathodes: A Critical Review

**DOI:** 10.3390/ma17020464

**Published:** 2024-01-18

**Authors:** Alessandro Dell’Era, Carla Lupi, Erwin Ciro, Francesca A. Scaramuzzo, Mauro Pasquali

**Affiliations:** 1 Department SBAI, Sapienza University of Rome, Via del Castro Laurenziano 7, 00161 Roma, Italy; francesca.scaramuzzo@uniroma1.it (F.A.S.); mauro.pasquali@uniroma1.it (M.P.); 2 Department ICMA, Sapienza University of Rome, Via Eudossiana 18, 00184 Roma, Italy; carla.lupi@uniroma1.it; 3 Department of Engineering Sciences, Guglielmo Marconi University, 00193 Rome, Italy; e.ciro@lab.unimarconi.it

**Keywords:** reticulated vitreous carbon (RVC), divalent ion electrodeposition, dimensionless analysis

## Abstract

In this paper, a critical review of results obtained using a reticulated vitreous carbon (RVC) three-dimensional cathode for the electrochemical depletion of various divalent ions, such as Cu^+2^, Cd^+2^, Pb^+2^, Zn^+2^, Ni^+2^, and Co^+2^, often present in wastewater, has been carried out. By analyzing the kinetics and fluid dynamics of the process found in literature, a general dimensionless equation, *Sh* = *f*(*Re*), has been determined, describing a general trend for all the analyzed systems regardless of the geometry, dimensions, and starting conditions. Thus, a map in the *log*(*Sh*) vs. *log*(*Re*) plane has been reported by characterizing the whole ion electrochemical depletion process and highlighting the existence of a good correlation among all the results. Moreover, because in recent years, the interest in using this three-dimensional cathode material seems to have slowed, the intent is to revive it as a useful tool for metal recovery, recycling processes, and water treatments.

## 1. Introduction

In recent years, a lot of research and studies in electrochemistry have been performed to enhance the performance of electrochemical devices by focusing attention on more advanced materials able to deliver or accumulate electrical energy at high and low temperatures. Batteries, fuel cells, or electrolyzers and supercapacitors have been extensively studied and analyzed [1,2,3,4,5,6,7,8]. New catalyst synthesis approaches for achieving high surface areas have been developed to produce nanoparticles, nanowires, or, in general, nanostructures as nanostructured thin film catalysts with extended surface areas, increasing significantly the electrochemical reaction activity [1,2]. Nanowires and nanopowders have also been synthesized having high electron storage densities and high diffusion rates and, thus, can be used to produce high-power sources [2]. Then, research has focused on developing new, cheaper, and more durable catalysts, by improving both the morphology and synthesis protocols, as alternatives to pure platinum, even if, normally, they present lower performances [3,4,5,6]. Generally, they are modified platinum-based catalysts containing other metals, such as Co, Cu, and Cr, or nonplatinum-based catalysts, such as non-noble metals, metal oxides, metal nitrides, and covalent organic frameworks, or, finally, organometallic catalysts [3,4,5,6,7,8]. Moreover, an increase in the performance of membranes or separators at high and low temperatures has been pursued, reaching lower ohmic losses and, thus, enabling higher current densities and higher efficiencies [3,4,5,6]. Several kinds of membranes or separators have been studied to work in different environments, allowing the use of less expensive catalysts. Finally, better engineering of the production processes of all the components present in these kinds of devices has enabled their widespread application [1,2,3,4,5,6,7,8]. 

Moreover, a glimpse has been provided into electrochemical technologies for electrical and/or electronic device waste recycling, for strategic material recovery, and for meeting the necessity to have a circular economy and low-environmental-impact processes, which, today, are becoming more important [9,10,11,12,13,14,15,16,17,18,19,20]. Herein, in particular, the aim of the authors is to show that an electrochemical technique can be effectively used for the electrochemical depletion of divalent metal ions from wastewater, utilizing a reticulated vitreous carbon (RVC) cathode, and to demonstrate how it is possible to give the general features of the electrochemical reduction process by a critical analysis of literature results obtained either by ourselves or other groups. For this purpose, the authors analyzed the dimensionless approach described in the literature for the depletion process to obtain a more general and exploitable empirical equation describing the electrochemical behavior of an RVC filter and the trend in the data, regardless of the chemical nature of the metallic divalent ions to be removed, geometry, fluid dynamics of the system, and different morphologies of deposits. The use of dimensionless analysis is justified by the reason that normally, the concept of scaling chemical process units can be applied in general to reactor systems, using a dimensionless group correlation [21]. To address scalability issues, the chemical processing industry often refers to the principle of similarity based on setting up the relationships existing between different scales of processing equipment [22,23,24,25]. Such relationships are usually determined through the so-called “dimensionless groups”, which characterize the process [26]. In this context, the well-known Buckingham theorem, regarding the relationship between the characteristic variables of a system, can be considered as a formal restatement of the requirement of dimensional consistency [27]. Generally speaking, dimensional analysis is considered as a prerequisite in the quantitative study of reacting systems, rate constants, transfer coefficients, and transport properties because it helps both in explaining the mechanisms associated with engineering processes and in developing an effective experimental design [28,29]. An electrochemical reaction can be carried out in the batch or continuous (mixed/plug flow) mode. In principle, reactors can operate with or without recycling, while further classifications are possible based on the flow arrangement (parallel/series flow) or electrical connections. The electrodes may be flat (two-dimensional solid electrodes) or porous (three-dimensional), either with a horizontal or vertical configuration [30]. The electrochemical methodology and reactors, especially when equipped with three-dimensional electrodes, have proved to be very efficient to decrease the amounts of metals in aqueous solutions [31,32,33,34,35,36,37,38,39,40,41,42,43,44,45] and have allowed the authors, in a previous study [46], to obtain a successful depletion of Ni^+2^ and Co^+2^. Considering that the hydrodynamic processes of ions transporting and reducing at reticulated vitreous carbon (RVC) three-dimensional cathodes are usually studied under mass-transfer control conditions, the dimensionless analysis is based on (i) the Sherwood number, which takes into account the convective and diffusive mass transports, and (ii) the Reynolds number, which considers the hydrodynamic condition of the flows passing through the RVC three-dimensional electrode.

A third dimensionless number, the Schmidt number, (Sc)*μD*^−1^, is also considered. A typical power expression, generally adopted to describe the mass transfer at porous electrodes, is as follows:

(*Sh*) = *m*(*Re*)*^n^*(*Sc*)^1/3^ (1)

The analytical equations for the Sherwood and Reynolds numbers are, respectively, as follows:

*Sh = k_m_εA_e_*^−1^*D*^−1^ and *Re = vμA*^−1^*A_e_*^−1^, where *k_m_* is the mass transport coefficient; *A_e_*^−1^ is the characteristic length; *D* is the diffusion coefficient, reported in Table 1 for the considered ions; *ε* is the RVC void volume fraction; *μ* is the solution kinematic viscosity, equal to about 0.011 cm^2^/s at room temperature; and *v* is the linear velocity.

## 2. Brief Comparison among Wastewater Treatment Techniques

Waste aqueous solutions containing low concentrations of heavy metal ions must be treated before discharging because, from a circular economy perspective, both the recovery/reuse of these metals can be achieved, and damage to the environment can be avoided. Several techniques for wastewater treatment, such as flotation, chemical precipitation, cementation, solvent extraction, ion exchange, and membrane filtration, are processes conventionally used in hydrometallurgy as leach liquor purification techniques [54]. However, the low concentration of metal ions in wastewater, compared to that in leach liquors, does not allow, from an economic point of view, the use of some of these techniques, as in the case of solvent extraction. However, the used organic solvents are flammable, volatile, partially water-soluble, and potentially toxic, with consequent environmental risks [55]. On the other hand, the use of chemical precipitation, although effective, is not selective and produces sludge containing hydroxides or sulfides of heavy metals, which are difficult to dispose, except in special landfills, with increasing operational costs [56,57]. One of the chemical cementation problems is that for presenting a high selectivity, a metal with a reduction potential very close to that of the metal to be recovered must be utilized as a reductive reactant, which involves very low process speeds [58]. The main problems in using membrane filtration are the requirement of a high investment cost and frequent membrane fouling, while the ion exchange method could work better with diluted metal solutions owing to the minor obstruction of the membranes or resins over time [59]. However, common ion exchange resins cannot perform well when utilized for the depletion of wastewater complex contaminants (such as the simultaneous presence of metal ions and organic pollutants), which could induce rapid exhaustion and even severe fouling, with the consequent significant reduction in the removal efficiency and increase in maintenance costs. However, all these recovery/removal methods present several drawbacks, such as the generation of large amounts of sludge, high reagent or energy requirements, incomplete removal of metal ions, difficulty in the treatment of large volumes of wastewater and, last but not least, high operational and maintenance costs. 

Lately, metal ion depletion processes, such as adsorption, photocatalysis, and electrochemical techniques, have attracted considerable interest. Adsorption provides high metal recovery capabilities with low operative costs; however, it presents low selectivity, and a large amount of waste production. Furthermore, adsorption needs frequent adsorbent regeneration [60,61]. Although photocatalysis does not produce sludge and instantly removes both metals and any organic pollutants present in wastewater, its industrial application is still limited, while it appears to be effective on a laboratory scale [62]. In this paper, the electrochemical technique, particularly electrodeposition on RVC, has been considered. It could be regarded as one of the most efficient techniques for the depletion of metal ions in wastewater because it does not produce sludge and can be automatically managed. However, this method is quite expensive, principally for the continuous flow of current that is required, if it is coupled with an analog technique able to directly recover metals in concentrated solutions on suitable support cathodes [20] after the electrochemical stripping of metals from RVC, which is a less costly and more efficient process that can be performed in the context of a circular economy and has a low environmental impact.

## 3. Materials and Methods

### 3.1. Theoretical Approach

Before discussing the dimensionless analysis, it is important to briefly describe the equations on which the process is based. The electrolysis rate can be expressed in terms of the reactant concentration, which decreases for a batch reactor containing a three-dimensional electrode as follows:

(2) $$c_{t}=c_{0}\cdot \exp^{\left(-\frac{k_{m}\cdot A_{e}\cdot V_{r}}{V_{t}}\right)\cdot t}$$

for the global process and is as follows:

(3) $$c_{out}=c_{in}\cdot \exp^{-\frac{k_{m}\cdot A_{e}\cdot V_{r}}{Q_{v}}}$$

for a single passage inside the reactor according to Equations (2) and (3).

**Figure 1:**
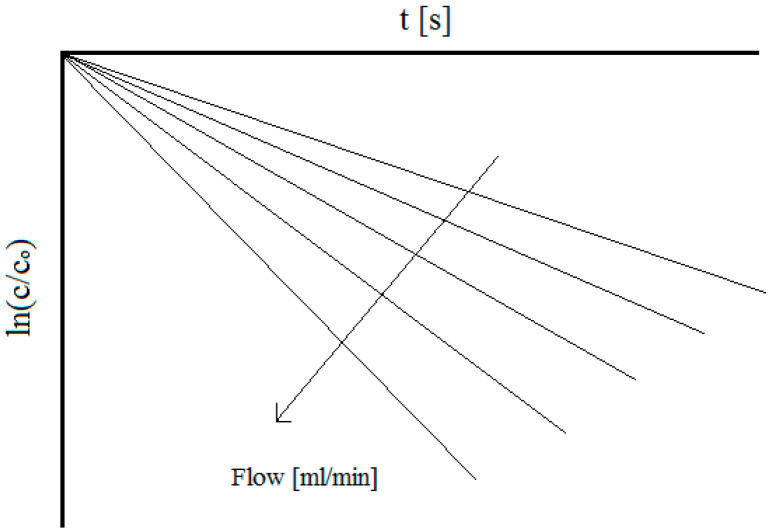
Trend of *ln(c/c_0_*) vs. time under mass transport conditions and at different flowrates for divalent metal ion depletion.

Equations (2) and (3) have been exploited for all the plug flow reactor (PFR) studies cited in this paper. By rearranging Equation (2) and plotting *ln(c/c_0_)* vs. time, it is possible to obtain lines like those reported in Figure 1, for which slope *m* is as follows:

(4) $$m=\frac{A_{e}\cdot k_{m}\cdot V_{r}}{V_{t}}$$

Under mass transfer control conditions, the value of *A_e_k_m_*, which increases with the flowrate, can be calculated to finally obtain *k_m_*, which is required for calculating the dimensionless Sherwood number. On the other hand, by knowing the cell geometry and the RVC porosity, the flow passage cross-section can be calculated and therefore, for each flow value, the linear velocity (*v*) and the dimensionless Reynolds number can be evaluated. 

**Figure 2:**
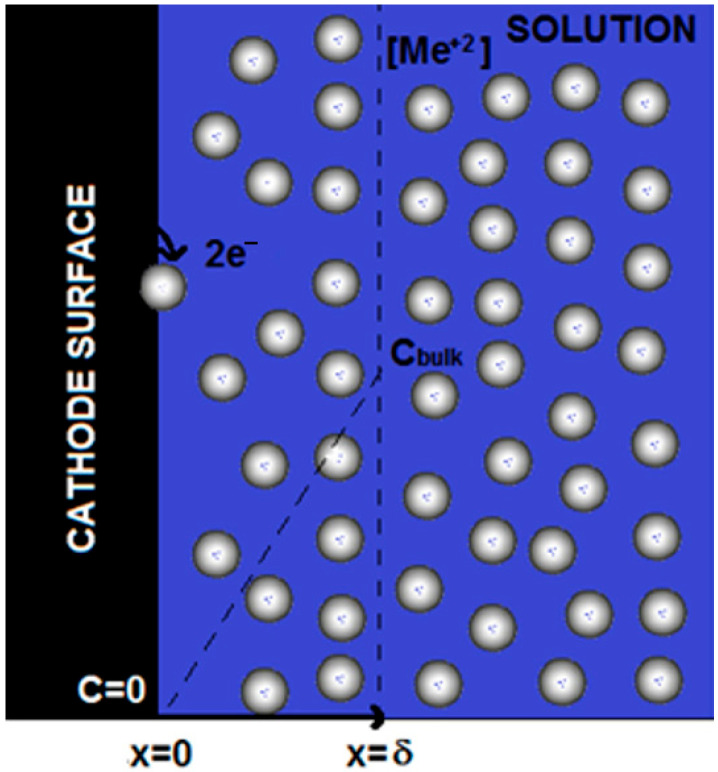
Mechanism for the ion electrodeposition.

As shown in Figure 2, the mechanism for the ion electrodeposition is similar for all the considered metals: When the mass control condition is established in the solution, the electrochemical reaction at the surface is so fast with respect to the mass transfer flow from the bulk that the ion concentration on the electrode surface becomes zero; and in proximity to the electrode surface, a diffusion layer is formed at the maximum concentration gradient and for which the thickness (*δ*) is a function of the flowrate. In particular, it decreases as the flow increases, enhancing the mass transfer coefficient and, consequently, the limiting current. To reach this situation, a suitable cathode potential must be imposed, as reported in Table 2. Under this condition, it is possible to use such dimensionless numbers to describe the process. In general, in eletrodeposition, the number of electrons that can be transferred in one or more steps can be evaluated by determining the charge transfer coefficient ( $\bar{\alpha }$ using Tafel analysis, but this is outside the scope of this paper; also, the charge transfer in this case does not represent the rate-determining step of the process.

Then, from the electrochemical point of view, to work under the mass transfer control condition means to stay under the limiting current density condition, for which the absolute value is as follows [44]:

(5) $$i_{L}=\frac{I_{L}}{A_{e}V_{r}}=nFk_{m}c_{t}$$

This condition is present when the electrochemical reaction kinetics on electrode surface are faster than the mass flow from the bulk to the electrode and in general, they are experimentally determined. Commonly, the kinetics are quantitatively related to the different ions through *c_t_*, while it is qualitatively bound to them through *n* and *k_m_*.

### 3.2. Experimental System Concept

**Figure 3:**
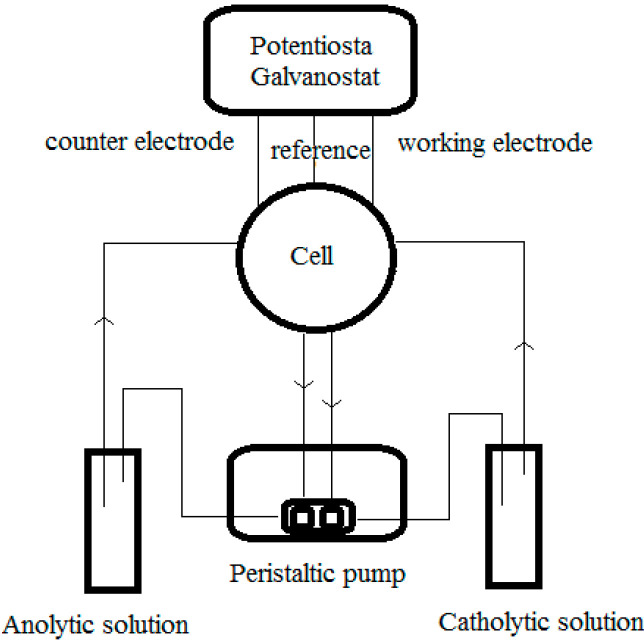
Schematic of the experimental apparatus described in all the papers considered in the present work.

**Figure 4:**
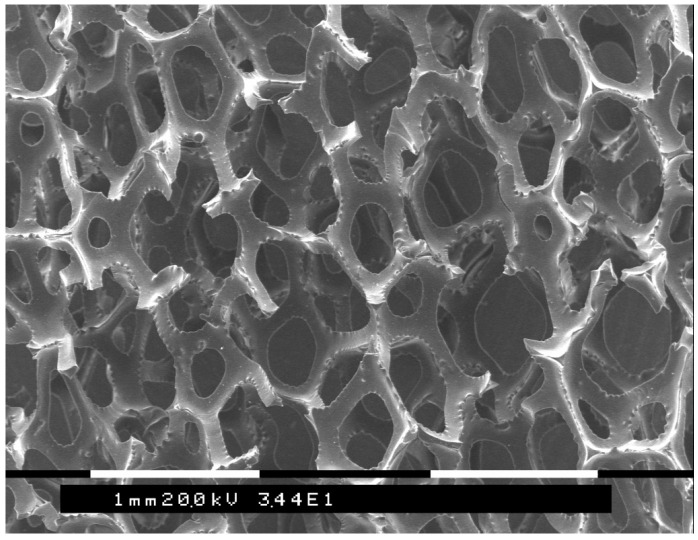
SEM image of an RVC cathode electrode (A_e_ = 27 cm^2^/cm^3^).

A generic schematic of the experimental apparatus described in all the papers analyzed herein is shown in Figure 3, while Figure 4 shows an example of the porous 3-dimensional RVC cathode on which the depletion process is based. Further details are reported in papers [31,32,33,34,35,36,37,38,39,40,41,42,43,44,45,46,50].

Despite the experimental setup being almost the same in all the cases taken into account, it seems that even slight differences, together with the peculiar features of each ionic species, can produce quite different results [31,32,33,34,35,36,37,38,44,46,50]. In principle, such differences can also be ascribed to the limited number of data, different characteristic length definitions, different morphologies of the deposits, and, in general, different systems that are used.

As far as the difference in the morphology is concerned, in general, it is possible to state that the morphologies of electrodeposited metals depend on several factors, such as the composition of the electrolyte, the current density, the temperature, the nature of the cathode support, fluid dynamics, and the addition of additives to the electrolyte, as well as the characteristics of metals and the interactions associated with the cathode materials [63,64,65].

However, to verify whether a general trend exists, a dimensionless analysis of the data presented in the literature has been carried out. Therefore, as aforementioned, the intention of this work is to try to overcome the differences found in the literature, thus obtaining a fair comparison of the results. In this sense, the present work can be considered as a critical review in the field of metal ion depletion from wastewater with an RVC cathode. Table 1 shows the geometric and dimensional features of the cells used in the experiments that were taken into account, together with the diffusion coefficient for each cation [35,38,47,48,49,51,52].

## 4. Discussion 

### 4.1. Nickel, Cobalt, and Zinc 

A previous dimensionless analysis for Ni^+2^ and Co^+2^ ion depletions under the mass control regime for different flowrates [46] was carried out, by the authors, considering a characteristic length (*A_e_*^−1^) equal to 27 cm^2^/cm^3^, a diffusion coefficient (*D*) of 0.6 × 10^−5^ cm^2^/s [60], a 97% RVC void volume (*ε*), a solution kinematic viscosity (*μ*) of 1.1 × 10^−2^ cm^2^/s, and an electrode geometry of 10.0 cm × 10.0 cm × 1.3 cm. The catholyte was a 0.1 M Na_2_SO_4_ solution, adjusted to pH 6 by the addition of H_2_SO_4_ and containing an initial concentration (*C_i_*) of Co^+2^ or Ni^+2^ equal to 150 ppm, while the anolyte was a 0.5 M Na_2_SO_4_ solution. The final Co^+2^ and Ni^+2^ concentrations in the solution were quantified using atomic absorption spectrometry and found to be less than 0.1 ppm in less than one hour. As expected, the *A_e_k_m_* values increased with the flowrate [46].

The current efficiencies ranged from 20% to 98% and from 3% to 45% for the nickel and cobalt depletions, respectively, and increased with the flowrate.

In this work, two different equations have been found for Ni^+2^ and Co^+2^ ion depletions on the RVC cathode as follows:

*Sh* = 0.077*∙Re*^0.78^*Sc*^0.33^ (6)

*Sh* = 0.207*∙Re*^0.293^*Sc*^0.33^ (7)

or considering the Schmidt number value, the relationship is as follows:

*log*(*Sh*) = 0.78∙*log*(*Re*) − 0.036 (8)

*log*(*Sh*) = 0.293∙*log*(*Re*) + 0.392 (9)

The flow range used for the depletion experiments was from 500 to 1300 mL/min in the case of Ni, while it ranged from 100 to 1650 mL/min in the case of Co. The linear speeds, instead, ranged from 0.66 to 1.70 cm/s and from 0.13 to 2.17 cm/s, respectively. Two different deposit aspects have been obtained. In particular, in the case of the nickel, the deposit appears homogeneous, compact, and uniformly distributed on the electrode, while in the case of the cobalt, it is spongy and dendritic. An anionic membrane was used between the cathode and anode.

Instead, other tests were carried out by Tan et al. [38] for Co^2+^ depletion. In this case, the initial concentration was 100 ppm, and the linear speeds ranged from 0.09 to 0.34 cm/s. In this system, instead of using a galvanostatic/potentiostatic source, a galvanic cell was conceived by coupling the Zn/Zn^+2^ redox couple, with a standard potential (E_0_) of −0.763 V, to a Co/Co^2+^ redox couple having a standard potential of E_0_ = 0.658 V on an RVC [41]. The catholyte was 0.2 M Na_2_SO_4_ and 0.1 M H_3_BO_4_ and contained the specified concentrations of Co^2+^, and the pH was adjusted to 4 by the addition of sulfuric acid or sodium hydroxide, while the anolyte was 0.1 M Na_2_SO_4_. The electrode geometry was 5.0 cm × 2.0 cm × 0.7 cm; also, in this case, an anionic membrane was used. Once the mass transfer control conditions were reached, more than 99% of the Co^+2^ was removed in less than two hours, as determined using atomic absorption spectrometry. The current efficiency was equal to about 82%, and the obtained metal deposit appeared particularly rough. The authors [38], in this case, reported different relationships in terms of A_e_k_m_ as a function of the linear velocity (*v*), as given by Equation (10), where parameters *a* and *b* are a function of A_e_ as follows:

*A_e_k_m_* = *a*∙*v^b^* (10)

As far as zinc ions are concerned, Lanza and Bertazzoli [50] focused on Zn^+2^ depletion either as a function of the flowrate or as a function of the electrode’s specific surface area. For this purpose, they started from a ZnCl_2_ solution with an initial zinc ion concentration of 54 ppm at pH 5.5 and using 0.1 M H_3_BO_4_ and 0.1 M KCl as the supporting electrolyte. RVC cathode electrodes with specific surface areas of 30, 40, 53, and 66 cm^2^/cm^3^ and a geometry of 15.0 cm × 5.0 cm × 1.25 cm [37] were used. 

The flow range was from 665 to 5300 mL/min, while the linear speed, instead, ranged from 1.7 to 14 cm/s when they used an RVC with a specific surface area of 53 cm^2^/cm^3^. The flow was kept constant at 2000 mL/mL when they changed the RVC specific surface area from 30 to 66 cm^2^/cm^3^.

A Nafion membrane was used between the cathodic and anodic compartments and for all the tests, a final concentration of less than 0.1 ppm was reached in less than one hour. The concentrations were measured using atomic absorption spectrometry. In particular, for those experiments, the authors did not report any particular dimensionless relationship between the Reynolds and Sherwood numbers, but they stated that good performance was obtained. Finally, the deposit quality was not mentioned.

### 4.2. Copper, Cadmium, and Lead 

On the other hand, Pletcher et al. [31] performed Cu^+2^ removal by working at different flowrates, with electrodes having specific surface areas equal to either 7 cm^2^/cm^3^ or 66 cm^2^/cm^3^, using the catholyte CuSO_4_ at an initial concentration of 10 ppm as a cation source. In this case, both the catholyte and anolyte were 0.5 M Na_2_SO_4_ solutions, kept at pH 2 by the addition of H_2_SO_4_, while the geometry of the electrode was 5.0 cm × 5.0 cm × 1.2 cm. The final concentration reached less than 0.1 ppm in a range of times from a few minutes to a few hours, depending on the RVC porosity. The authors did not describe the quality of the copper deposits, but they stated that good efficiency was achieved. In this case, a different definition of the characteristic length was taken into account. The authors defined *d_e_* as follows:

(11) $$d_{e}=\frac{4A}{P}=\frac{2BS}{\left(B+S\right)}$$

where *A* is the cross-sectional area of the flow, *P* is the wetting perimeter of the cross-section, while *B* and *S* are the width and thickness of the RVC, respectively.

Using the above-mentioned *d_e_* definition, the authors found the following dimensionless relationship among the Sherwood, Reynolds, and Schmidt numbers:

*Sh* = 2.7*∙Re*^0.48^*Sc*^0.33^ (12)

or considering the Schmidt number value, the relationship is as follows:

*log*(*Sh*) = 0.48∙*log*(*Re*) + 1.53 (13)

However, it is worth stressing that the values of *log*(*Re*) and *log*(*Sh*), for our purpose, were recalculated using the definition of *d_e_* used in this work, namely *A_e_.*

The flow range used in the experiment was from 420 to 7000 mL/min, while the linear speed ranged from 1.2 to 20 cm/s for the considered cases.

On the other hand, Podlaha and Fenton [32] performed Cu^2+^ reduction using an RVC electrode with a specific surface area of 27 cm^2^/cm^3^ and a geometry of 10.2 cm × 4.5 cm × 0.87 cm. The concentrations of the Cu^2+^ copper ion ranged from 10^−4^ M to 10^−2^ M in a 0.8 M NaSO_4_ supporting electrolyte solution kept at pH 3 upon the addition of H_2_SO_4_. A Nafion membrane was used between the cathodic and anodic compartments. The electrolyte flowrate through the RVC electrode was varied between 0.012 and 2.6 cm/s (2.8–620 mL/min). In this case, the obtained deposit thus had a granular morphology. 

The copper concentration was determined spectrophotometrically, using a wavelength of 560 nm. In this work, the authors found the following dimensionless relationship for Cu^+2^ ion depletion on the RVC cathode, using *A_e_* as the characteristic length:

*Sh* = 0.44*∙Re*^0.69^*Sc*^0.33^ (14)

or considering the Schmidt number value, the relationship is as follows:

*log*(*Sh*) = 0.69∙*log*(*Re*) + 0.74 (15)

Moreover, by always considering Cu^+2^ ions, Y.P. Hor and N. Mohamed [33] instead operated a galvanic cementation system. In this system, instead of using a galvanostatic/potentiostatic source, a galvanic cell was conceived by coupling the Fe/Fe^+2^ redox couple, with a standard potential (E_0_) of −0.44 V, to a Cu/Cu^2+^ redox couple having a standard potential of E_0_ = 0.34 V. Therefore, in this case, the copper reduction occurs spontaneously on an RVC cathodic support. The authors used an RVC electrode with a specific surface area of 53 cm^2^/cm^3^ and a geometry of 5 cm × 2 cm × 1 cm and an anion exchange membrane. The electrolyte solutions were pumped by a peristaltic pump into the cell flowing from the bottom to the top of the cell. The initial concentration of the Cu^2+^ copper ion was 100 ppm in 0.5 M H_2_SO_4_. The flow range used in the experiment was from 2 to 20.1 mL/min, while the linear speed ranged from 0.016 to 0.160 cm/s, values which were much lower with respect to those obtained by other authors.

The concentrations of the effluent were determined using an absorption spectrophotometer. During operation in the recycling mode, they reached almost 100% Cu^+2^ removal, showing that reticulated vitreous carbon is a very good electrode material for copper ion removal. In this work, the authors found the following dimensionless relationship for Cu^+2^ ion depletion on the RVC cathode, using *A_e_* as the characteristic length for defining the Reynolds and Sherwood dimensionless numbers:

*Sh* = 0.94*∙Re*^0.63^*Sc*^0.33^ (16)

or considering the Schmidt number value, the relationship is as follows:

*log*(*Sh*) = 0.63*∙log*(*Re*) + 1.07 (17)

Considering Cd^+2^ ions, Llovera-Hernandez et al. [34] performed depletion by working in the galvanostatic mode with a de-aerated electrolyte and an electrode with 5.0 cm × 5.0 cm × 1.0 cm size and an RVC specific surface area of 40 cm^2^/cm^3^. The electrolyte composition was different for different experiments. Only the results for the 0.5 molar concentrations of NaCl and Na_2_SO_4_ were considered, with an initial pH value equal to 6 and an initial Cd^+2^ ion concentration of 225 ppm. The flowrate and the linear speed were 4600 mL/min and 16 cm/s, respectively. The final Cd^+2^ concentration was in the range of about 2–10 ppm, which was reached in about 2 h, and an electrochemical method (differential pulse-stripping voltammetry) was used to measure the Cd^+2^ concentration in the solution. A Nafion membrane was used between the anodic and cathodic compartments. A constant current density equal to 3.5 A/m^2^ was used under mass transport control conditions. The authors did not report any relationship between the Reynolds and Sherwood dimensionless numbers. So, in this case, the data were used to calculate them.

Furthermore, Dutra et al. [35] performed tests, in the potentiostatic mode, for Cd^2+^ removal, using a de-aerated electrolyte and an electrode having a specific surface area of 40 cm^2^/cm^3^ and a geometry of 12.0 cm × 5.0 cm × 1.2 cm. The anolyte and catholyte were 0.5 M Na_2_SO_4_, adjusted to pH 2 upon the addition of H_2_SO_4_. Starting from 210 ppm of Cd^2+^ dissolved in the catholyte, a final concentration of 0.1 ppm, as measured using atomic absorption, was obtained in about one hour and a half. A protonic membrane (Nafion) was used to separate the anodic and cathodic compartments. The metallic deposit thus obtained was also, in this case, nodular [35,39]. The authors did not report any dimensionless relationship. In any case, the best test results were obtained using a flowrate and a linear speed of 4200 mL/min and 12 cm/s, respectively.

Finally, considering Pb^+2^ ions, depletion tests were carried out by Bertazzoli et al. [36] and Widner et al. [37] by operating at three different flowrates ranging from 1000 to 4000 mL/min, with electrodes having specific surface areas equal to 53, 37, 27, and 11 cm^2^/cm^3^ and a geometry equal to 15.0 cm × 5.0 cm × 1.25 cm. 

A protonic membrane (Nafion) was used in the experimental setup, and a mixed solution of 0.5 M H_3_BO_4_ and 0.05 M KNO_3_ at pH 4.8 was used as the supporting electrolyte. Starting from a 50 ppm initial Pb^2+^ concentration, obtained using Pb(NO_3_)_2_, a final concentration of less than 0.1 ppm was obtained in a time range from about 20 min to 2 h, depending on the RVC porosity and flowrate. In this case, the current efficiencies ranged from 4.6% to 22.3% and from 1.8% to 14.1% for 90% and 99% Pb^+2^ removals, respectively, and it increased with increasing specific electrode surface (*A_e_*) of the RVC.

As for Cu and Co, a rough lead deposit was obtained. Also, in this case, like Pletcher, the authors used Equation (11) as the characteristic length and found the following dimensionless relationship [36]:

*Sh* = (24 ± 7)*Re*^(0.32±0.05)^*Sc*^0.33^ (18)

or considering the Schmidt number value, the relationship is as follows:

*log*(*Sh*) = (0.32 ± 0.05)∙*log*(*Re*) + (2.28 ± 0.12) (19)

Nevertheless, as before, *log*(*Re*) and *log*(*Sh*), for our intent, were recalculated using *A_e_* as the characteristic length.

In addition, other tests have been also carried out by C. Ponce de Leon and D. Pletcher [44] for Pb^2+^ depletion at six different flowrates in a chloride solution, using RVC cathodes with different specific surface areas and having a geometry equal to 5.0 cm × 5.0 cm × 1.2 cm. The flow range used for the tests was from 60 to 6050 mL/min, while the linear speed ranged from 1.6 to 16 cm/s. A 0.5 M NaCl solution at pH 2 was used as the supporting electrolyte. Starting from a 7 ppm initial Pb^2+^ concentration, a final concentration of less than about 0.5 ppm was obtained in less than 60 min, working at a current efficiency of about 80% when 90% of the Pb^2+^ had been removed. The concentrations were determined using atomic absorption spectroscopy. In this case, the authors found *k_m_* values, using the voltammetric technique to determine the limiting current for different flows and different RVC cathodes, and using Equation (5).

These results are in agreement with those obtained by other authors in other experiments. Also, in this case, the authors reported several relationships similar to Equation (10), with different *a* and *b* coefficient values, depending on the specific RVC electrode surface (*A_e_*) that was used.

Moreover, for lead and copper ions, respectively, the authors found the following dimensionless relationships among the Sherwood, Reynolds, and Schmidt numbers, using Equation (11) as the characteristic length:

*Sh* = 5.6*∙Re*^0.44^*Sc*^0.33^ (20)

for Pb^+2^ ion depletion in a solution of 0.5 M NaCl at pH 2 and a 60 ppi (40 cm^2^/cm^3^) RVC and

*Sh* = 2.4*∙Re*^0.48^*Sc*^0.33^ (21)

for Cu^+2^ ion depletion in a solution of 0.5 M Na_2_SO_4_ at pH 2 and a 10 ppi (7 cm^2^/cm^3^) RVC, or considering the Schmidt number values, the respective relationships are as follows:

*log*(*Sh*) = 0.44∙*log*(*Re*) + 1.48 (22)

and

*log*(*Sh*) = 0.48∙*log*(*Re*) + 1.8 (23)

Finally, the authors concluded that electrolytic treatment using a reticulated vitreous carbon cathode could be usefully applied to the waste stream to decrease the ion level to a concentration suitable for direct discharge. 

To maximize the process results, it is necessary to work under mass transport control conditions, i.e., at the limiting current value, which depend on the ion concentrations in the bulk, flowrates, and temperatures. In every case, it is necessary to determine the cathodic potentials beyond which the mass control condition is present, such as those found in the literature and reported in Table 2. With some exceptions, most authors worked in the potentiostatic mode. 

It is worth stressing that they could be different because these cathodic potential values depend on different parameters and always have to be experimentally determined. This means that when working under galvanostatic conditions (as in the case of Llovera-Hernandez et al. [34]), a current that implies those cathodic potential values must be set up. Straying too much toward more cathodic potentials, for example, by increasing the value of the current too much, means decreasing the process efficiency because other reactions, such as hydrogen evolution, may occur. In all the cases, either a protonic membrane (Nafion) [31,32,34,35,37,50] or an anionic membrane [29,33,46] was used between the cathodic and anodic compartments.

## 5. Dimensionless Analysis

**Figure 5:**
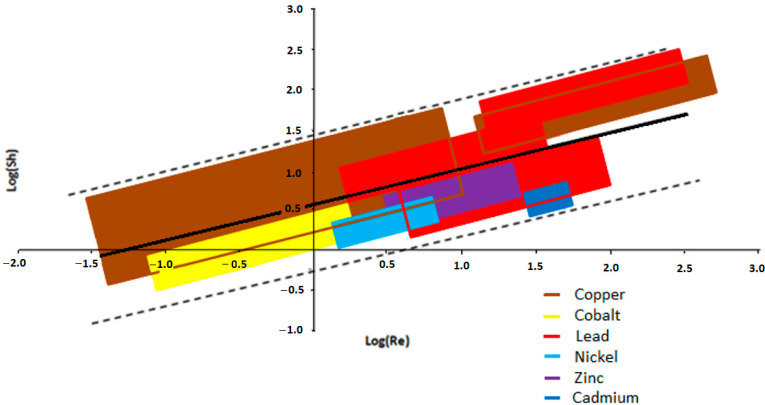
Recalculated trend of log(Sh) vs. log(Re) equations for the depletions of Ni^+2^ [46], Co^+2^ [38,46], Cu^+2^ [31,32,33,44], Cd^+2^ [34,35], Pb^+2^ [36,37,44], and Zn^+2^ [50].

For comparing the literature results, it is necessary to point out that the definition of the characteristic length is crucial because it has a drastic effect on the coherence of the results. Actually, even though some authors, like Pletcher and Bertazzoli, used different definitions [31,36,37,50], the use of the *A_e_*^−1^ term seems to be the most appropriate because it takes into account the RVC porosity (in terms of the number of pores per inch, ppi), which can also be different for cathodes with the same geometry. For a fair comparison, using the literature results and *A_e_*^−1^ as the characteristic length, pairs of dimensionless numbers were calculated and evaluated for all the examined and cited works, and Figure 5 shows where the calculated pairs of dimensionless numbers fall in the *log*(*Sh*) vs. *log*(*Re*) plane more frequently for each different divalent ion if this kind of experiment is carried out. This clearly shows a general trend in a well-defined region of the graph. 

The statistical analysis highlights a strong correlation among the experimental results as well. Indeed, from Table 3, where some statistics, such as the standard deviations (*σ_x_* and *σ_y_*), covariance (*Cov_xy_*), Pearson correlation coefficient (*r*), and degrees of freedom (number of pairs minus two), are reported, it is possible to notice that the *r* value (0.71) exceeds the critical one, *r_c_* (0.174), highlighting a strong correlation.

This behavior can be best described by the equation of the straight black line shown in Figure 5, for which the coefficients were calculated by minimizing the error, using the least square method, of the difference between the experimentally measured and theoretically calculated points, using Equation (24) as follows:

*log*(*Sh*) = 0.47∙*log*(*Re*) + 0.57 ± 0.85 (24)

In Equation (24), ±0.85 represents the region of the graph, in Figure 5, delimited by the dotted lines, where the totality of the points falls. In summary, if Equation (11) is used as the equivalent diameter for the dimensionless numbers, the constant term of the resulting *Sh* vs. *Re* equation is too high, and the curve, in general, does not fall in this region; however, if the specific area of RVC (*A*^−1^) is used instead, even presenting, in some cases, quite different slopes and constant terms, the curves lie between the two dotted lines in all the cases. This does not mean that Equation (11) cannot be used but only that to compare the data, it is very important to use the same equivalent diameter definition, whatever it is, but in our opinion, *A*^−1^ is more suitable. Moreover, it is worth stressing that in our opinion, if further experiments will be considered, the sizes of the colored regions could be extended and overlap with each other but very probably would remain between the dotted lines all the same. Finally, all these considerations obviously are not valid, for example, if different kinds of electrodes (other than RVC), different types of solvents (other than water), or other additives in the electrolyte solutions (besides those mentioned in this work) are considered.

## 6. Conclusions

A suitable analysis of the literature results related to the kinetics and fluid dynamics of the depletion processes of different divalent metal ions from wastewater allowed a fair comparison among the different outcomes obtained by the authors considered in this work. This, in turn, allowed the identification of a particular region in the *log*(*Re*) vs. *log*(*Sh*) plane, where it is more probable to find these outcomes in terms of such dimensionless numbers. The discussion and description of such results highlighted that the electrochemical technique using the RVC cathode material should have more consideration as it can be taken into account as an efficient tool for wastewater treatment in the removal of divalent metal ions. The outcomes suggest that if the same dimensionless number definition is used, general and similar behaviors for all the described systems could be found regardless of the differences in geometry, dimensions, and initial conditions. Thus, a general dimensionless trend equation has been proposed to describe the electrochemical depletion of such divalent ions from wastewater, using an RVC cathode. The statistical analysis highlights a good correlation. The obtained empirical equation can be exploited, first and foremost, for designing an RVC electrochemical filter for the divalent ion depletion process.

## Figures and Tables

**Table 1:** Dimensional cellular features and diffusion coefficients for divalent ions undergoing electrochemical depletion, as described in various reference papers.

Ion	RVC Section (cm^2^)	*A_e_* (cm^2^/cm^3^)	Diffusivity (cm^2^/s)	Schmidt Number (Sc)	Reference(s)
Zn^+2^	6.5	27–40–53–66	1.2 × 10^−5^ [47,48,49] Chloride solution	917	[50]
Cu^+2^	6	7–66	0.49 × 10^−5^ [31,51,52,53] Sulfate solution	2200	[31]
	4	27	0.45 × 10^−5^ [51,52,53] Sulfate solution	2200	[32]
	2	53	0.5 × 10^−5^ [51,52,53] Sulfate solution	2200	[33]
Cd^+2^	5	40	0.71 × 10^−5^ [39] Sulfate solution	1550	[34]
	6	40	0.71 × 10^−5^ [39] Sulfate solution	1550	[35]
Pb^+2^	6.5	11–27–37–53	2.0 × 10^−5^ [36] Nitrate solution	550	[36,37]
	6	7–40–66	0.7 × 10^−5^ [44] Chloride solution	1570	[44]
Co^+2^	1.5	53	0.65 × 10^−5^ [51] Sulfate solution	1830	[38]
	10	27	0.65 × 10^−5^ [51] Sulfate solution	1830	[46]
Ni^+2^	10	27	0.6 × 10^−5^ [51] Sulfate solution	1830	[46]

**Table 2:** RVC cathodic potentials (referenced to SCE) required to reach mass transport control conditions, as found in the literature.

Ion		References
Ni^+2^	−1100 mV vs. SCE	[46]
Co^+2^	−1200 mV vs. SCE	[46]
Zn^+2^	−1400 mV vs. SCE	[50]
Cu^+2^	−500 mV vs. SCE	[31]
Cd^+2^	−850 mV vs. SCE	[35]
Pb^+2^	−800 mV vs. SCE	[36,44]

**Table 3:** Statistical data: standard deviations (σ_x_ and σ_y_), covariance (Cov_xy_), Pearson correlation coefficient (r), critical Pearson coefficient (r_c_), alpha level (α), degrees of freedom (fd).

σ_x_	σ_y_	Cov_xy_	r	r_c_	α	fd
0.88	0.58	0.37	0.71	0.174	0.05	125

## Data Availability

Data are contained within the article.

## Nomenclature

*A_e_* is the three-dimensional electrode’s specific surface (cm^−1^)
*A_e_*^−1^ is the characteristic length (cm)
*c_t_* is the concentration at time *t* (mol/cm^3^)
*D* is the diffusion coefficient (cm^2^/s)
*F* is the Faraday constant
*k_m_* is the mass transport coefficient (cm/s)
*L* is the RVC cathode length (cm)
*Q_v_* is the volumetric flowrate (cm^3^/s)
*n* is the ion valence
*N* is the number of recirculations
*N_cells_* is the number of cells
*S* is the RVC cathode section (cm^2^)
*t_r_* is the reactor residence time (s)
*t_f_* is the total depletion time (s)
*V_r_* is the three-dimensional electrode’s volume (cm^3^)
*V_t_* is the solution volume (cm^3^)
*v* is the linear velocity (cm/s)
*ε* is the RVC void’s volume
*μ* is the solution’s kinematic viscosity (cm^2^/s)
*α* is the alpha level
*σ_x_* and *σ_y_* are the standard deviations
*Cov_xy_* is the covariance
*r* is the Pearson correlation coefficient
*r_c_* is the critical Pearson coefficient
*fd* is the degrees of freedom
